# A Survey of Horse Selection, Longevity, and Retirement in Equine-Assisted Services in the United States

**DOI:** 10.3390/ani11082333

**Published:** 2021-08-07

**Authors:** Ellen M. Rankins, Carissa L. Wickens, Kenneth H. McKeever, Karyn Malinowski

**Affiliations:** 1 Rutgers Equine Science Center, Department of Animal Sciences, Rutgers, The State University of New Jersey, New Brunswick, NJ 08901, USA; mckeever@sebs.rutgers.edu (K.H.M.); karynmal@njaes.rutgers.edu (K.M.); 2 Department of Animal Sciences, University of Florida, Gainesville, FL 32611, USA; cwickens@ufl.edu

**Keywords:** horse, therapeutic riding, EAAT, EAS, behavior

## Abstract

**Simple Summary:**

The aim of this study was to provide information about horses and ponies in equine-assisted services (EAS), particularly in the areas of selection, longevity, and retirement as little published information exists about this sector of the United States horse industry. Survey results revealed centers do use selection procedures when evaluating horses which often included initial screenings and trial periods. Horses are active in programs from anywhere less than a year to over 20 years. The greatest number of horses are active for 1–6 or 7–10 years. Horses are retired for a variety of reasons. The most common reasons include unsoundness, behavior, and other health issues. We conclude behavior, soundness, and health are key considerations when selecting and retiring horses in EAS. These areas should be focused on at an individual horse level in future research efforts. The information presented in this article helps us understand the horses in EAS programs, provides a starting point for future research, and begins to explore the unique needs of programs and horses in EAS.

**Abstract:**

Little published information exists on the horses in equine-assisted services (EAS), particularly their selection, longevity, and retirement. The purpose of this study was to characterize horses and procedures used in EAS. A pilot survey was developed using focus group discussions and distributed to Professional Association of Therapeutic Horsemanship, International (PATH Intl) centers in Florida (*n* = 45, Part I) before further modification and distribution to members of PATH Intl., American Hippotherapy Association (AHA), eagala, and Certified Horsemanship Association (CHA) (*n* = 26,000, Part II). Response rates were 36% (Part I) and 0.7% (Part II). Centers report a median of 10 (Part I) or 9 (Part II) horses and ponies. Selection procedures included initial screening (Part I = 100%, Part II = 96%), pre-purchase or pre-donation exam (I = 64%, II = 60%), acclimation period (I = 100%, II = 84%), trial period (I = 91%, II = 90%), and other (II = 11%). Horses remained active in programs for less than a year to over 20 years with the greatest number working 7–10 (Part I) or 1–6 (Part II) yr. In Part I of the study, behavior (44%) was the leading cause of retirement followed by unsoundness (33%). In Part II, unsoundness was the highest ranked response followed by behavior. Behavior, soundness, and health emerged as key factors in horse selection and retirement. Future work should focus on investigating these issues at an individual horse level.

## 1. Introduction

Equine-assisted services (EAS) encompass therapy (psychotherapy, physical therapy, occupational therapy, speech therapy, and counselling) equine-assisted learning (in education, professional development, and organizational development) and adapted equestrian activities (therapeutic or adapted riding, adapted equestrian sports, interactive vaulting, adapted driving, and horsemanship). Many of these programs serve participants facing emotional, social, cognitive, physical, or a combination of challenges. Until recently, EAS was known as equine-assisted activities and therapies (EAAT in the United States). Discontinuation of the term EAAT was recently recommended in the culmination of a consensus building process [1].

The American Horse Council added EAS to the category of working horses in their 2017 Economic Impact Report [2]. This addition and an increasing number of accredited centers over the years provides evidence that there is growing interest in and recognition of EAS. The Professional Association of Therapeutic, Horsemanship (PATH Intl.) known at the time as the North American Riding for the Handicapped Association (NARHA) accredited thirty-nine centers in 1976 [3]. The number of accredited centers increased to a total of six hundred ninety-two centers in 2005 and to eight hundred seventy-three in 2017 [4,5].

Despite the growth and increasing recognition of EAS little information exists about these programs and the equids engaged in this work meaning further information is needed. Some organizations, such as PATH Intl., publicly report information about the centers and professionals the organization accredits and certifies [5]. There is often little information provided about the equids engaged in the work.

High horse turnover and behavioral issues may be a common problem in EAS based on anecdotal evidence and preliminary work [6]. The incidence of these challenges is unknown. Additionally, the general lack of published literature regarding programs and equids in EAS means we know very little about the unique challenges and concerns this sector of the equine industry faces.

The identification of challenges and information to inform future research and decision making is critical for the EAS industry. Expanding our understanding of the horses and other equids in EAS will allow for more informed decision making to ensure horse well-being. More information can also inform future research studies investigating horse welfare status, an area which continues to receive increasing scrutiny. Behavioral reactions, often in the form of reaction to a stimulus, are recognized as a leading cause of accidents in equestrian sports [7,8,9]. Characterizing horses in EAS and the incidence of behavioral issues can help protect the people, whether they be practitioners and instructors or clients and participants.

The purpose of this study was to investigate the current state of horse selection, longevity, and retirement in EAS in the United States while providing basic demographic information on the programs and their equids. The results are intended to begin the process of characterizing the EAS industry and its equids to inform future research and education efforts.

## 2. Materials and Methods

This study was undertaken in two parts (Part I and Part II). Part I focused on the development and piloting of a survey to collect information on horses in EAS. Part II involved the distribution of the survey and response collection at a national level in the United States.

Part I of the study was conducted in Florida. Study procedures and instruments were approved by the University of Florida Institutional Review Board. A fifty-nine-question survey was developed in Qualtrics^®^ (version 12.2016, Qualtrics, Provo, UT, USA). Question development was guided by focus-group discussions. EAS professionals residing outside of Florida were identified based on the authors’ network of contacts. These individuals participated in focus-group discussions where their responses to a series of questions were recorded using a digital voice recorder (WS-852, Olympus America, Inc., Center Valley, PA, USA) [10,11]. The discussion was subsequently transcribed and key themes identified for development of a questionnaire [10,11]. The questionnaire was then reviewed by equine industry professionals at the University of Florida for clarity and then pre-tested by these individuals. Suggested changes were implemented and the process repeated to produce the final questionnaire (Table A1) used in Part I of the study. The questionnaire was divided into three sections: center and staff demographics (twelve questions); horse selection, retirement, and management (thirty questions); and horse and personnel training (seventeen questions). Question types included fill-in-the-blank, open-ended, multiple choice, and Likert-type scale responses. It should be noted that the survey was developed and distributed prior to the recent publication of recommendations for optimal terminology in the United States [1]. Terms used to describe the programming and services offered by centers reflects current use at the time of distribution and not the recent recommendations for optimal terminology.

The study was distributed via email to the executive directors, volunteer coordinators, and barn or horse managers at forty-five PATH, Intl. (Denver, CO, USA) accredited centers in the state of Florida. A public listing of centers is maintained by PATH Intl on their website. This list was used to identify the target population (PATH Intl. centers in Florida) for Part I of the study. A unique identifier was assigned to each center and the list of centers and assigned identifiers subsequently destroyed to retain response anonymity. The unique identifier was automatically recorded by the Qualtrics^®^ software when a survey response was initiated. This approach allowed multiple responses from the same center to be grouped together. Emails were sent every two weeks in a repeat contact strategy over an eight-week period in January and February of 2017 [12]. In the email, we requested the executive director, barn or horse manager, and volunteer coordinator complete the questionnaire. This approach was employed as each center had only one email address listed on the website, and it was generally unknown which individuals had access to the listed email address. Three centers had two staff members who responded to the survey. The small number of responses from multiple staff members at a single center prevented comparison among staff member’s description of their program. All responses were retained for subsequent analysis.

Study procedures and instruments for Part II of the study were approved by the Rutgers University Institutional Review Board. A nine-question survey was developed in Qualtrics^®^. The survey used was a modification of the survey developed in Part I of the study. The questionnaire in Part I was designed to cover a broad array of topics. Following response collection, key areas of interest were identified as horse selection, longevity, and retirement. While other areas of interest exist, the need to shorten the survey in hopes of attaining a higher response rate was a key consideration. Questions pertaining to these issues were retained in Part II. Question formats were changed to elicit a more nuanced view of the horses at a center rather than the general trends at a center. For an example, rather than asking respondents to choose the average age of the horses at their center, respondents were asked to indicate the age of all horses in the program. Questions retained were related to the activities offered at the center, the number of horses and ponies active in the program, animal age, workload, sources from which animals were acquired, selection procedures, and reasons for retirement. It should be noted that the survey was developed and distributed prior to the recent publication of recommendations of optimal terminology in the United States [1]. Terms used to describe the programming and services offered by centers reflects current use at the time of distribution and not the recent recommendations for optimal terminology. Respondents were asked to consider the general trends in their program when completing the survey. Question types included fill-in-the-blank, multiple choice, sliding scale, and ranking (Table A2). A link to the survey was distributed via email and social media platforms by the American Hippotherapy Association, Inc (AHA, Inc., Fort Collins, CO, USA); PATH, Intl; eagala (Santaquin, UT, USA), and the Certified Horsemanship Association (CHA, Lexington, KY, USA) to their members. Survey distribution and response collection began in October of 2019 and concluded in June of 2020. A repeat contact strategy with four contacts spaced 2–4 weeks apart was used for survey distribution [12]. Each organization distributed the survey link and instructions directly to their members. Responses were collected anonymously. Each organization was sent a separate link to determine how many members from each organization completed the survey.

Responses from Parts I and II were exported to Microsoft Excel^©^ (version 2102, Microsoft Inc., Redmond, WA, USA) for sorting. Responses from Part I of the study were summarized in Excel© using frequency counts, descriptive statistics, and qualitative responses. In Part II of the study, ranking responses with non-consecutive responses or initial ranking of greater than 1 were renumbered such that ranking began with 1 and proceeded consecutively to the highest-ranking category. Text responses provided for “other” were grouped into like categories where appropriate for further analysis and reporting. The total number of activities offered in the program and total number of selection procedures were calculated by summing the number of responses selected for the question. Weighted averages of horse age and length of time animals remained in the program were calculated based on the percentage of animals in a category and the average number of years represented by the category for use in further analyses. (Example: A center with 25% of its animals between 1 and 5 years of age, 25% of its animals between 6 and 10 years of age, and 50% of its animals between 10 and 15 years of age would have a weighted animal age of 15.25 [3 × 0.25 + 8 × 0.25 + 13 × 0.5 = 15.25].)

Statistical analyses were performed in SAS (v 9.4, Cary, NC, USA). Descriptive statistics were computed using the univariate procedure. Kendall’s Correlation with a Bonferroni correction was used to analyze the correlation of reasons for retirement and longevity in program with selection practices, workload, and activities offered. Variables influencing reasons for retirement were further explored using a canonical discriminant analysis with the most frequent reason for retirement as the classifier and variables selected using stepwise discriminant analysis. Canonical discriminant analysis is an approach used with multivariate data to separate classes in a lower dimensional space. The dependent variable (most frequent reason for retirement) is a categorical variable, and many possible independent variables were considered. This combination of factors made canonical discriminant analysis an appropriate and logical choice for data analysis. A generalized linear model with a Gaussian distribution was used to analyze the effect of the most frequent reason for retirement, and donation and free lease as sources of animals on the weighted average of longevity in the program. Variables for inclusion in the model were selected using LASSO. Significance was set at *p* < 0.05.

Box plots present the 10th, 25th, 75th, and 90th percentiles of the data as the boxes and whiskers. The median is presented in a solid line and the mean in a dashed line. All data points falling outside of the 10th and 90th percentiles are presented as individual data points. Data are presented as medians with an interquartile range (IQR).

## 3. Results

### 3.1. Part I

#### 3.1.1. Response Rate

Nineteen completed responses from sixteen centers were received from the forty-five centers contacted for a response rate of 36%. One incomplete response was received and removed from the data set.

#### 3.1.2. Staff and Center Demographics

Thirty-seven percent (*n* = 7) of respondents serve in one role at the center while the remainder of respondents serve in two (*n* = 5) or three (*n* = 7) roles. Respondents indicating they only served in one role served as the executive director of the center. Executive directors (*n* = 12), barn or equine managers or horse resource managers (*n* = 7), volunteer coordinators (*n* = 4), instructors (*n* = 10), volunteers (*n* = 2), and program administrators, managers, or directors (*n* = 3) responded to the survey.

Certifications held by the respondents included PATH Intl. Registered Level Therapeutic Riding Instructor Certification (*n* = 14), PATH Intl. Therapeutic Driving Instructor Certification (*n* = 3), PATH Intl. Equine Specialist in Mental Health and Learning Certification (*n* = 1), Board Certified Behavior Analyst (*n* = 1), veterinarian (*n* = 1), and volunteer administrator (*n* = 1). Two respondents indicated they held no certifications.

Respondents worked or volunteered at centers which are PATH Intl Member centers (68%) or PATH Intl, Premier Accredited Centers (32%). The number of years centers had been members of PATH Intl. ranged from 1–5 years to 36–40 years with the largest number (*n* = 6) of respondents indicating their center had been a member of PATH Intl. for 1–5 years (Table 1).

One and a half (0.25–3) equine related occurrences per year were reported with responses ranging from 0 to 22. An equine related occurrence was defined as an event that results in, or nearly results in, injury or danger to a person or persons and involves an equid [13].

One hundred (72.5–375) participants are served each year by centers with the number of participants ranging from 10 to 1994. Only one respondent indicated participants received services for free.

Respondents indicated their centers had 10 (7–14) horses active in the program with the number of horses ranging from 4 to 17. Additionally, one respondent indicated the center had one donkey and two respondents indicated their centers had Miniature Horses (two and one, respectively). The total number of equids reported on was 199.

**Figure 1:**
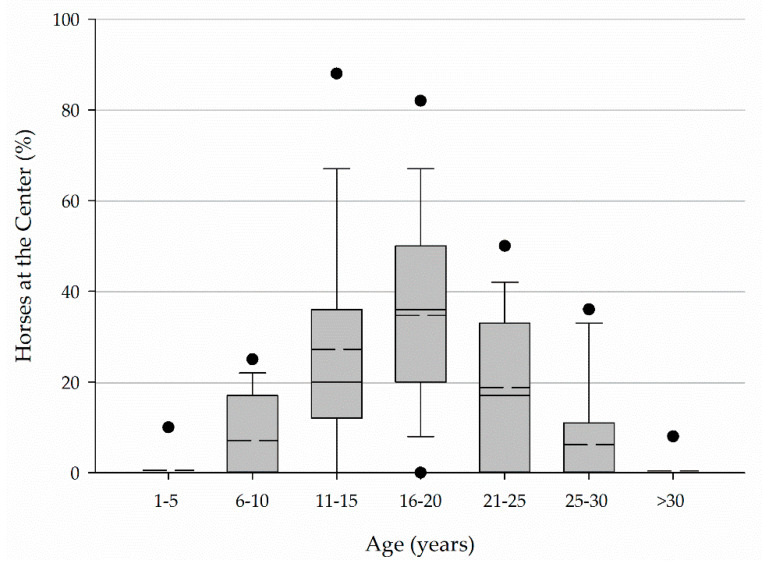
Age of horses at Florida PATH Intl centers.

Horses ranged in age from 1–5 years to over 30 years with the greatest percentage of horses at a center falling between 16 and 20 years of age (Figure 1).

**Figure 2:**
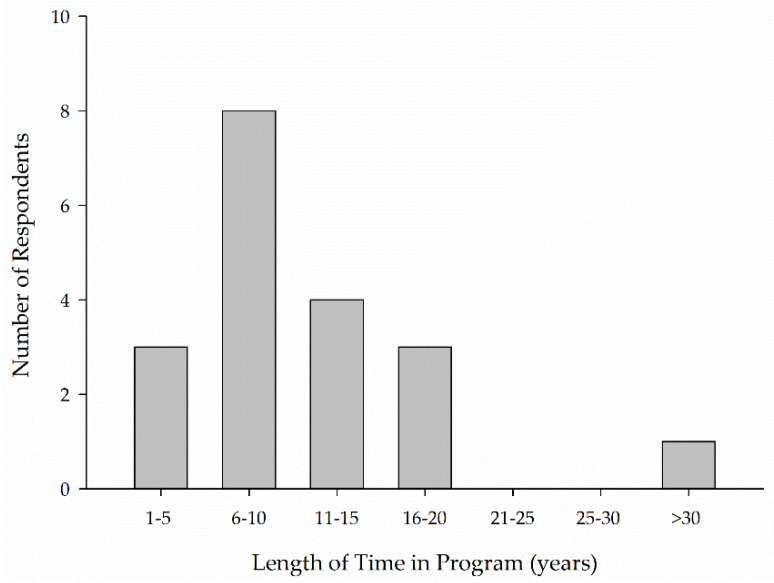
Length of time, on average, horses are active in a center’s program.

Horses remained, on average, active in a center’s program for anywhere from 1–5 years (*n* = 3) to over 30 years (*n* = 1) with the greatest number of centers having horses active for 6–10 years (*n* = 8) (Figure 2).

The amount of land available to centers ranged from 1–5 acres (*n* = 5) to over 25 acres (*n* = 4). Other acreages available to centers included 6–10 acres (*n* = 3), 11–15 acres (*n* = 3), 16–20 acres (*n* = 2), and 21–25 acres (*n* = 2).

#### 3.1.3. Horse Selection, Retirement, and Management

Respondents indicated their centers acquired horses through donation (89% of respondents), purchase from trainers or breeders (58%), purchase from private owners (47%), adoption from rescues (42%), free leases where the previous owner retains ownership and the center makes no payment of a lease fee (21%), paid lease (5%), and exchange for reduced board (5%). Ninety percent of respondents indicated their center had a protocol that was used in selecting horses. Staff felt very prepared (95%) or prepared (5%) to perform the selection and evaluation protocol. Selection procedures included: an initial screening of the horse (100%) an acclimation period upon arrival at the center (100%), a trial period (91%), and a pre-purchase or pre-donation exam by a veterinarian (64%). Acclimation periods lasted 4–6 days (18%), 7–9 days (27%), 10–12 days (9%), 13–15 days (18%), or 28–42 days (27%). Trial periods lasted 3–4 weeks (50%), 7–8 weeks (10%), or 12–13 weeks (40%).

Respondents were asked about the characteristics they considered when selecting horses in an open-ended question. The following characteristics were considered desirable: brain; heart; high tolerance level; teachability; soundness; accepting of unbalanced riders; accepting of sidewalkers; enjoys human contact; calmness; friendliness; cooperation; ability to walk, trot, and canter; quietness; well-broke; kind disposition; slow and smooth movement; low reactivity; soft eyes; easy to handle, responsive to cues; quiet demeanor; good health; good hooves; loads into trailer; stands tied; willing; and previous show experience. Undesirable characteristics included: dangerous behaviors (i.e., biting, kicking, rearing, bucking), cribbing, stable vices, spookiness, previous injuries, and fearfulness. General characteristics considered included: disposition, conformation, movement, temperament/personality, age, health, mind, body, spirit, size, gender, previous history, condition, behavior, fitness, attitude, and temper.

Upon retirement horses go to a private owner (56%) or stay at the center (44%). Fifty-eight percent of respondents indicated their center had criteria to determine when a horse should be retired. Staff felt very prepared (84%), prepared (11%), or neither prepared or unprepared (5%) to use the criteria. The retirement criteria included the horse’s behavior during lessons (100%), the horse’s interactions with participants (100%), the horse’s interactions with volunteers (100%), soundness (100%), overall health (100%), and age (11%). Behavior (44%), unsoundness (33%), age (11%), and death (11%) were the primary reasons for retiring horses. When asked about the specific behavioral reasons for retiring horses, respondents listed aggression, biting or nipping, lameness, personality changes, health, back issues, pinning ears, and kicking.

Ninety-five percent of centers were responsible for the daily care and management of horses. Another entity provided for the daily care and management of the horses at the other 5% of centers. Turnout is provided in the form of pasture (67%), a small grass lot or paddock (11%), or a dry lot (22%). All horses are turned out with another horse or group of horses. In the spring and summer, horses are turned out 5–8 h (33%), 9–12 h (22%), 17–20 h (11%), or 20–24 h (33%) a day. In the fall and winter, horses are turned out 5–8 h (22%), 9–12 h (33%), 17–20 h (11%), or 20–24 h (33%) a day. The average horse at a center had a Body Condition Score of 4 (12%), 5 (38%), 6 (38%), or 7 (12%) out of 9. One respondent indicated they did not know what a Body Condition Score was. Two centers had horses who cribbed. No other stereotypic behaviors were reported.

**Figure 3:**
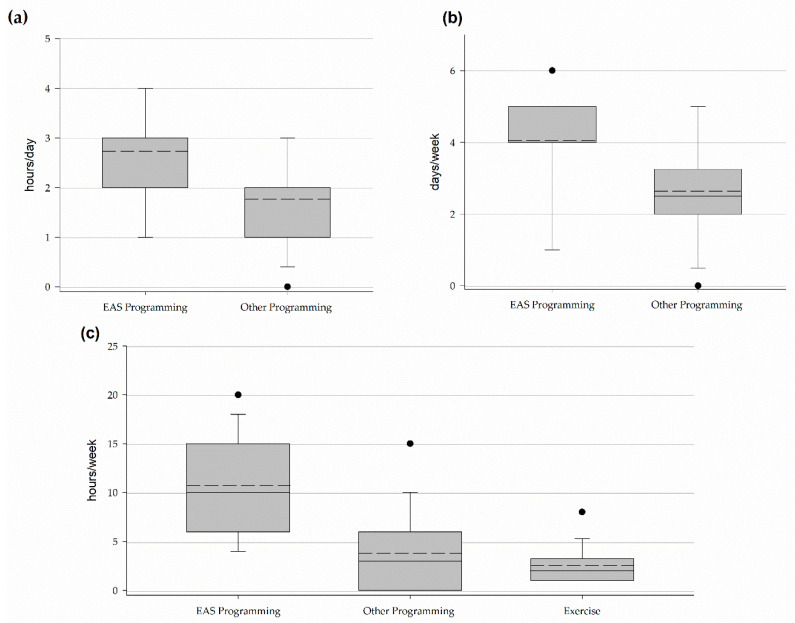
Workload of horses in Florida PATH Intl. centers. EAS programming includes adaptive or therapeutic riding, vaulting, driving, equine assisted therapy, and equine assisted learning. Other programming includes such things as summer camps and lessons for riders without disabilities. Exercise includes structured exercise and conditioning activities administered by trained personnel. (**a**) Hours per day (**b**) Days per week (**c**) Hours per week.

Centers offer and horses participate in therapeutic riding (100%), equine assisted learning (79%), summer camps (68%), riding lessons for riders without disabilities (58%), equine assisted psychotherapy (37%), hippotherapy (16%), and adapted driving (16%). Centers offered, on average, 4 types of programming with a range of 2–7 types of programming. Horses work 3 (2–3) hours a day 4 (4–5) days a week for a total of 10 (6–15) hours per week in EAS programming (Figure 3). In addition, horses work 2 (1–2) hours a day 2.5 (2–3.25) days a week for a total of 3 (0–6) hours a week in other program activities (Figure 3).

One program indicated they did not exercise horses outside of program activities while the remaining respondents indicated horses were exercised outside of program activities. Horses are exercised 2 (1–3.25) hours a week by riding (100%), lunging (67%), hand-walking (33%), ground driving (17%), and groundwork (6%) (Figure 3). The exercise is administered by instructors (83%), barn, horse, or horse resource managers (72%), volunteers (67%), executive directors (33%), volunteer coordinators (11%), schooling teams (11%), able-bodied riders (6%), or exercise riders (6%). Schooling teams, able-bodied riders, and exercise riders were write-in responses provided under other and are provided as direct quotations of responses. Sixteen percent of respondents indicated their horses only participated in program activities or exercise. Other activities outside of program activities and exercise included trail rides (58%), free lunging (26%), clinics and workshops (5%), round penning (5%), and rodeo (5%). Five percent of respondents indicated their horses did not participate in human-horse interactions outside of program activities. The remaining 95% indicated their horses participate in grooming outside of preparation for program activities (95%), spending time with people in the pasture or stall as the person observes or pets the horse (68%), desensitizing (5%), and playing in a sensory trail (5%).

#### 3.1.4. Horse and Personnel Training

Center staff were very prepared (63% and 68%), prepared (32% and 32%), or neither prepared or unprepared (5% and 0%) to train new horses and horses already in the program. Thirty-two percent had a horse training program all staff were required to use with progress tracking occurring through a computer program or written record. Twenty-one percent sometimes use a horse training program and tracking system. The remaining 47% have a training program where staff keep mental notes and report progress verbally to the center leadership. Respondents were asked to describe their training programs using the terms listed in Table 2.

Fifty-eight percent of respondents used negative reinforcement in weekly training and 33% used it in correcting unwanted behaviors. Seventy-five percent used positive reinforcement in weekly training and 67% used it in correcting unwanted behaviors. No respondents used negative punishment. Twenty-five percent used positive punishment in weekly training and 42% used it in correcting unwanted behaviors. Thirty-three percent used systematic desensitization in weekly training and 50% used it in correcting unwanted behavior. Seventeen percent of respondents also chose other when describing their horse training program. These respondents defined other as “Taking the Lead” and natural horsemanship.

Thirty-seven percent of respondents did not use the help or services of outside trainers. The remainder occasionally (42%) or regularly use outside trainers (21%). Those using the services of an outside trainer use the help to correct unwanted behaviors among program horses (75%), provide education or training to staff and/or volunteers (33%), train some of the horses entering the program (8%), and proactive training (8%).

Eight percent of respondents indicated they had no challenges with their horse training program, while the remaining ninety-two percent had moderate challenges. When asked to describe the challenges, responses included (as written by respondents): communicating with team members on the ways in which we are training and why they are a benefit to the herd; time; individual horses having their own set of bad manners, insecurities, etc.; inexperienced handlers; number of handlers in general; consistency among handlers; and handlers recognizing the correct behavior and rewarding it appropriately. Respondents thought the industry does not need better trained horses (17%) or better horse training programs (11%), some centers need better trained horses (56%), or better horse training programs (56%), or the industry needs better trained horses (28%) or horse training programs (33%).

Center staff are very prepared (74%) or prepared (26%) to train their volunteers in horse handling techniques. Ninety-five percent of centers have a program for training volunteers while the remaining 5% do not. Eighty-four percent of respondents indicate their center offers continuing education for staff and volunteers.

Fifty-eight percent of respondents indicated they would use instruction regarding horse training techniques and programs while the remaining forty-two percent might use such instruction. Sixteen percent of respondents were interested in using outside resources (presenters or curriculum) in continuing education for staff. Seventy-five percent of respondents were interested in using outside resources in continuing education for staff and volunteers. The remaining five percent were not interested in using outside resources for continuing education. Those interested in using outside resources in continuing education would be interested in having a workshop or clinic at their center (100%), attending an off-site workshop or clinic (67%), participating in an online course (83%), or using curriculum (78%).

### 3.2. Part II

#### 3.2.1. Response Rate

One hundred seventy-six consenting completed survey responses were received from 26,000 potential respondents for a response rate of 0.68% (Table 3). Two non-consenting responses and 189 incomplete responses were also received and removed from the data prior to subsequent analysis.

#### 3.2.2. Staff and Center Demographics

**Figure 4:**
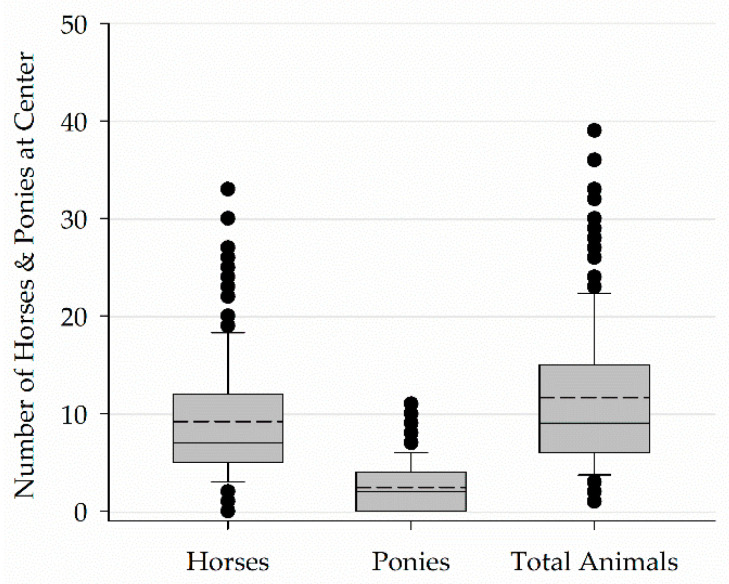
Number of horses and ponies in centers offering EAS programming.

Respondents reported seven (5–12) horses and two (0–4) ponies were active in their programs for a total of nine (6–15) animals active in a program (Figure 4).

**Figure 5:**
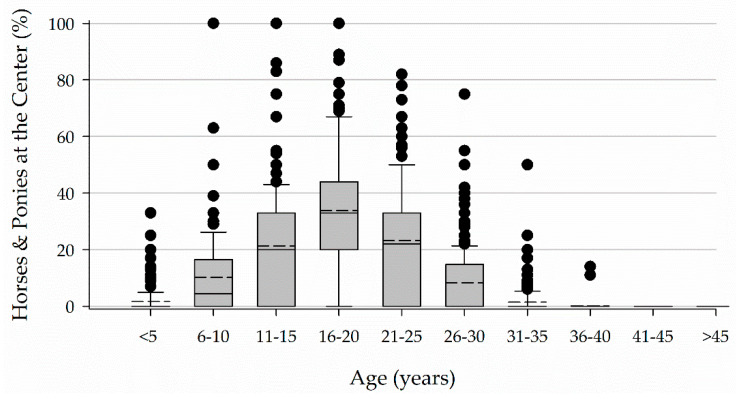
Age of horses and ponies.

Horses and ponies ranged in age from less than 5 years to 36 to 40 years with the greatest percentage (33, 20–44%) falling between 16 and 20 years of age (Figure 5).

**Figure 6:**
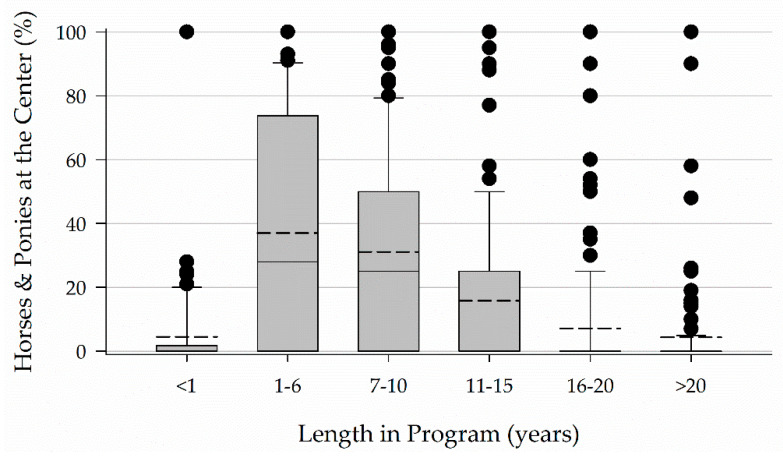
Length of time horses and ponies remain active in a program.

Horses and ponies spent anywhere from less than a year to over 20 years in a program. Respondents reported the greatest percentage (28, 0–73.75) of horses and ponies in their programs stayed active for 1 to 6 years (Figure 6).

#### 3.2.3. Horse Selection, Retirement, and Management

**Figure 7:**
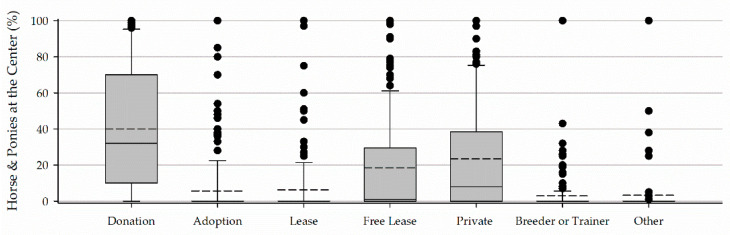
The percentage of horses and ponies at a center acquired from donation, adoption from rescues (adoption), lease, free lease, purchase from private owners (private), purchase from breeder or trainer (breeder or trainer), and other sources.

Horses and ponies were acquired through donation (32, 10–70% of horses and ponies at a center), adoption from rescues (0, 0–0%), lease (0, 0–0%), free lease (1, 0–29.5%), purchase from private owners (8, 0–38.5%), purchase from breeders and trainers (0, 0–0%), and other sources (0, 0–0%) (Figure 7). Other sources listed by respondents included won in a contest, public sales or auctions, not applicable, direct rescue, and owned by staff or facility.

Procedures used in selecting horses and ponies included an initial screening (96% of respondents), a pre-purchase or pre-donation exam by a veterinarian (60%), an acclimation period at the center (84%), a trial period (90%), and other selection procedures (11%) (Table 4). Other selection procedures listed included ongoing training, prior occupation or use, a quarantine period, questionnaires and videos, Lyme titers, activity specific habituation, and behavioral and disposition testing.

**Figure 8:**
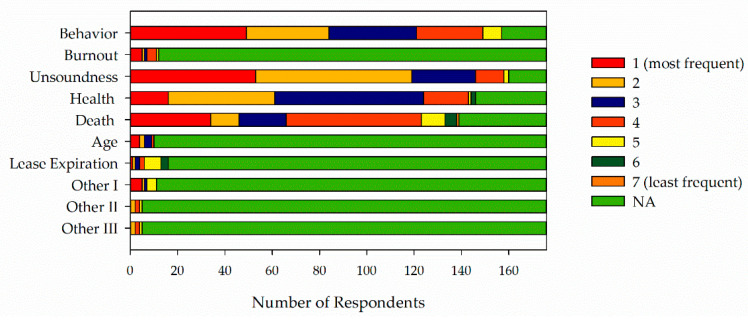
Reasons for retiring horses and ponies. Reasons for retirement were ranked consecutively from 1 (most frequent) to least frequent. Respondents could choose NA if the reason was not applicable in their situation.

When asked reasons for retiring horses and ponies, respondents ranked (1 = most frequent; 7 = least frequent) age (2, 1–3;), behavior (2, 1–3), and unsoundness (2, 1–3) as the most frequent reasons followed by other behavioral reasons including burnout (2.5, 1–4), other health issues (3, 2–3), death (4, 2–4), lease expiration (5, 3.5–5), and other reasons (Figure 8). Other behavioral reasons including burnout, age, and lease expiration were categories created from the grouping of similar write-in responses listed under other. Other reasons included horses being sold, earning retirement, move to another barn, length of time in program, need for other activities, change of location, scheduling issues, foaling, size or movement do not fit program needs, and career change.

Horses participate in adaptive or therapeutic riding (79% of respondents), equine-assisted physical therapy (40%), equine-assisted occupational therapy (39%), equine-assisted speech therapy (21%), equine-assisted psychotherapy (34%), equine-assisted learning (52%), adaptive driving (14%), interactive vaulting (5%), traditional riding lessons (3%), adaptive unmounted activities (3%), and other activities (4%) (Table 5). Traditional riding lessons and adaptive unmounted activities are write-in responses in other which occurred multiple times and thus, were grouped together. Other activities included competition showmanship, Paralympics, vocational programming, recreation, education, and sport. Centers offer, on average, three types of programming with a range of 1–8 types of programming.

**Figure 9:**
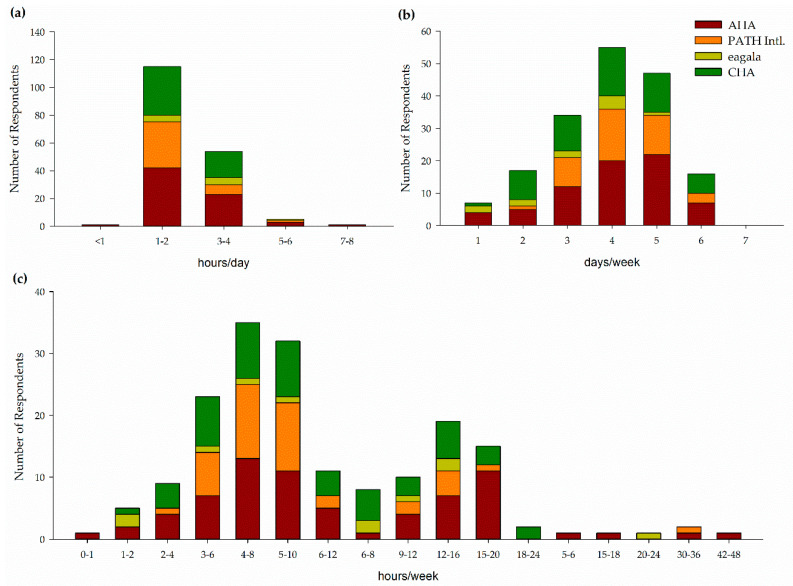
Workload of horses and ponies in AHA, PATH Intl, eagala, and CHA centers. (**a**) Hours per day (**b**) Days per week (**c**) Hours per week.

Horses work anywhere from less than an hour a day to 7–8 h a day 1 to 7 days a week for a total workload between 0–1 h per week and 42–48 h per week (Figure 9).

#### 3.2.4. Relationships among Variables

Reasons for retirement and length of time horses spent in the program were not significantly correlated with selection practices, workload, or activities offered.

Donation as a means of acquiring horses (*p* = 0.0032) and the most frequent reason for retirement (*p* = 0.0007) were significant predictors of length of time horses spent in the program. Donation as a means of acquiring horses predicted horses would spend 0.04 (*p* = 0.0032) more years in the program. If the most frequent reason for retiring horses was death, horses were predicted to spend 4.15 (*p* < 0.0001) more years in the program.

**Figure 10:**
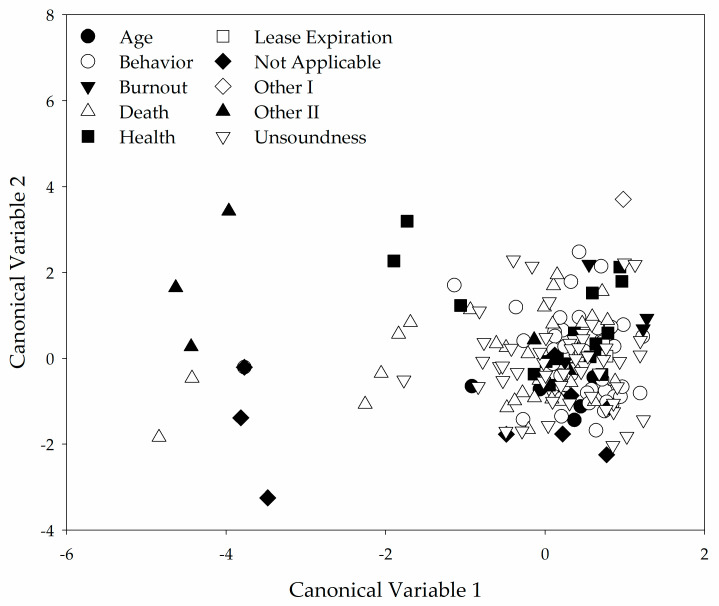
Plot of canonical variables 1 and 2 derived from the canonical discriminant analysis. Little grouping by classification variable is observed.

The canonical discriminant analysis used the most frequent reason for retiring a horse (age, behavior, burnout, death, health, lease expiration, not applicable, other I, other II, and unsoundness) as the classification category. A significant (*p* < 0.0001) Pillai’s Trace value (1.20) indicated there were detectable differences across the classification groups. Canonical variable 1 had the greatest squared canonical correlation at 0.30 which is below the suggested cutoff value of 0.4 [15]. Canonical variable 1 explained 29% of variance with an Eigenvalue of 0.45. The low discriminatory power of the identified canonical variables is supported by the absence of distinct groups when the canonical variable values are plotted against one another (Figure 10).

## 4. Discussion

In Part I of the study, a response rate of 36% was achieved. This response rate is comparable to other surveys conducted in the EAS industry as PATH Intl reported a 21% response rate in an employment analysis, and Watson and colleagues reported a response rate of 40.7% in their survey of PATH Intl centers to document horse care and use [16,17]. While these response rates are lower than desired, this may be the response rate which is achievable for this population. The small number of centers with multiple staff members responding to the survey may have been influenced by the relatively large number (63%) of respondents who serve in multiple roles and the contact strategy utilized. Using the email address listed on the PATH Intl website for each center allowed for access to potential respondents through a public avenue but meant the recipient of the email was unknown. Thus, it is possible that the recipient of the email was the only staff member to complete the survey. The email asked that the survey link be forwarded to other staff members who served in the roles of executive director, barn/equine manager, and/or volunteer coordinator, however, there is no way of knowing whether this request was honored. The large proportion of respondents with a PATH Intl Registered Level Instructor Certification is as expected as the largest number of certifications awarded each year are at the registered instructor level [18].

A much lower response rate of 0.68% was achieved in Part II of the study. Part of this low response rate may be attributable to the method of contact. AHA was the only organization which included the invitation to participate and survey link in a stand-alone email rather than including it in a regular communication such as an e-newsletter and had the highest response rate at 2.8%. The number of potential respondents could be inflated as individuals could be members of more than one association. Possible overlap was not accounted for in the numbers and response rates reported as we did not have access to membership lists for each organization. It is also possible for individuals not employed in EAS or not involved in horse care and use to be a member of one or more of the organizations. Even considering these factors, the response rate for Part II of the study was very low creating limitations in how the data should be interpreted and used. Results should also be interpreted with the differences between the two questionnaires and broad focus of the questionnaire in Part I being kept in mind. Careful consideration of these issues should be addressed in pre-testing for future surveys.

In Part I, 68% of centers were PATH Intl Member Centers and the remaining 32% were PATH Intl Premier Accredited Centers. These numbers align closely with numbers published by PATH Intl wherein they indicated 68% of centers are Member Centers and the remainder are Premier Accredited Centers [5]. Assuming an even distribution of centers, these results indicate an unbiased sampling of the target population and indicates other results are likely representative of the target population.

The greatest number of centers had been in operation for 1 to 5 years. This short length of operation indicates there are centers which have recently opened and are joining this sector of the equine industry. This finding could be an indicator of continued industry growth or a high level of center turnover.

Great diversity is observed in EAS as the number of participants served, number of horses active in the program, and types of programming offered varied between centers across Parts I and II of the study. The number of horses and ponies active at centers are similar to numbers reported elsewhere [5,17]. Most centers offered more than one type of programming indicating individual horses may work in a variety of programming types, although this was not specifically asked in either of the surveys. Use of horses in multiple types of programming provides support in favor of the need for a unifying term which has been recently discussed [1]. Some differences in the types of programming offered by centers in different organizations were observed in Part II of the study, although these patterns are based on numerical differences and not statistical differences. These patterns are unsurprising given the mission of each organization and the types of training and certification offered by each. While specialization and separate areas of expertise are apparent, there is also considerable overlap present in the data set.

Horses between 16 and 20 years of age represented the greatest percentage of horses at a center based on the median for each age category. This pattern was consistent across Parts I and II of the study. The commonness of horses between 16 and 20 years of age supports the idea of many horses in EAS being on a second or third career. Anecdotal evidence has suggested that this is most often the case. This older age and associated previous experience may be a desirable characteristic as previous experience was considered when selecting horses.

Six to ten years was the most common response for the number of years horses are typically active in a program in Part I of the study, while 1–6 years encompassed the greatest percentage of horses in Part II of the study. This difference across Parts I and II of the study may be due to differences in how the question was asked. In Part I, respondents were asked to choose the answer that best represented the average length of time horses spent in their program. In Part II of the study, respondents indicated the percentage of horses in their program that remained active for the listed number of years. Changing the way in which the question was asked allowed for collection of more nuanced data, but limits comparison across parts of the study. Considering a typical lifespan for the horse is 25–30 years or longer this working lifespan in EAS seems relatively short. However, when the age of the horses in the program is also considered, this working lifespan seems more reasonable. The age of and length of time horses remain in a program should be considered in conjunction. Anecdotal evidence of EAS as a second or third career and the reported horse age indicate horses in EAS may present a biased sample. This bias should also be borne in mind when considering other results.

The leading source of horses as reported in these data sets is donation, aligning with results from a recently published survey [17]. In this article, Watson and colleagues suggest this may be due in part to the limited funding programs have available to purchase or lease horses [17]. The authors also point out this is very similar to reports on university programs [17,19]. The disparity between the existence of selection and retirement protocols and staff preparedness in performing these activities seen in Part I, and the high prevalence of donation for horse acquisition point towards a lack of standardization and protocols. Working from personal experience rather than a professional protocol creates communication and standardization problems such as the ones documented by Anderson and colleagues in relation to horse temperament [20].

As expected, some common themes emerged from the selection criteria, including size, age, temperament or personality, movement, soundness, and experience. The identification of common themes in selection criteria provide areas for future research. Further research could assess whether these traits correlate with or predict success in EAS and help identify the most effective methods for evaluating these traits. The small list of ill-defined undesirable characteristics may also warrant further research.

In Part I of the study, all respondents indicated behavior during lessons, interactions with participants, soundness, and overall health are used to determine when a horse should be retired. The importance of the horse’s behavior, soundness, and health in determining an appropriate retirement point suggests these should also be of importance when selecting horses. The importance of these factors in determining when to retire a horse also suggest these as areas for further research as a greater understanding of the variables affecting these factors may aid centers in developing protocols and managing horses in a way that ensures their longevity in a program.

Behavior was the leading cause of retirement in Part I of the study while unsoundness was the leading cause of retirement in Part II, followed closely by behavior. Unsoundness and behavior are intricately interconnected as unsoundness or lameness can be detected via behavior observation [21,22]. Without accurate diagnostic work, it is possible that horses classified as being retired because of behavior issues are being retired because of an underlying unsoundness or health issue that has manifested itself as a change in behavior. Future work should take this issue into consideration. We would suggest providing definitions for the terms used or allowing respondents to describe the reason for retirement. Inconsistent use of terms and disagreement over definitions presents a major limitation in this study and will continue to be a concern unless addressed in future studies.

The behaviors of concern listed in Part I of the study, which included biting or nipping, kicking, and pinned ears, are similar to behaviors of concern listed in other reports [6]. This commonality suggests these behaviors should be of interest when monitoring horses in EAS and evaluating their welfare. The appearance of a burnout category in Part II of the study is surprising given that respondents were provided with a category of behavior and then chose to write-in and rank another behaviorally assessed trait such as burnout or changes in disposition as another reason for retirement. The appearance of this category warrants further research. While burnout has been researched and discussed in humans, there does not appear to have been any work conducted on this topic in horses. Further research should be aimed at identifying what burnout is in horses and whether it can be accurately and reliably assessed. Watson and colleagues in their study reported a lower incidence of health issues among horses engaged in EAS when compared with other sectors of the equine industry [17]. This finding raises the question of why unsoundness and health issues are reported as leading causes of retirement for horses in EAS.

The workload of horses was comparable to the workload reported by Watson and colleagues and well below the maximum workload provided by PATH Intl in their standards for accreditation [13,17]. Time spent in EAS programming does not tell the full story. In Part I of the study, respondents reported horses were active during exercise (2 h per week), other activities or human-horse interactions, and other program activities such as traditional riding lessons (3 h per week). Future assessments of horse workload in EAS need to consider these other sources of work. In horses, workload is based primarily on physical exertion [23]. In EAS, where physical exertion is low it may be necessary to consider the cognitive load horses experience. In humans, cognitive load has been linked to energy expenditure [24]. Deficits in balance and muscular functioning among clients in EAS can impact the horse’s way of going which may in turn further increase the workload of horses even when working at slow gaits such as a walk [25].

The low level of staff preparedness in training volunteers and horses despite having protocols in place indicates provision of protocols in these areas does not ensure staff preparedness. Since much of horse training has traditionally been based on negative reinforcement, it is interesting to note that positive reinforcement was the most common form of training utilized and was followed by negative reinforcement [9]. The authors of this paper do note that with increasing public awareness, there has been a shift towards more use of positive reinforcement in horse training [9]. Thus, several possible explanations exist for the observed results: this sector of the industry adopting the practice of positive reinforcement more quickly than the rest of the industry, more widespread use of positive reinforcement than previously thought, or respondents mistakenly identifying their training practices. Previous survey work has resulted in differing levels of success in having respondents correctly define training techniques, however, in the present study the terms were defined for respondents [26,27]. Correct definitions do not always result in correct identification of the technique in a real-world situation [27]. Also, of interest is the inclusion of two responses in the other category that were listed as natural horsemanship and “Taking the Lead”. As with all training methods, these methods still rely primarily on operant conditioning and yet, respondents felt these needed to be noted separately. Training seems to be an area where needs exist as most respondents indicated a need for centers or the industry to have better trained horses and horse training programs. Additionally, most centers have moderate challenges in their horse training programs with time, consistency, knowledge, and inexperience being some of the common challenges.

EAS is a sector of the equine industry that is open to extension and other educational programming as most respondents were interested in or might be interested in instruction regarding horse behavior and training techniques and other continuing education resources. Clinics and workshops at a center were the preferred venue followed by online courses, curriculum, and off-site workshops and clinics.

The lack of significant correlations among variables and low number of significant predictor and classification variables in the analyses undertaken points towards a need for further research to guide selection and management procedures for optimal horse retention and welfare. In this study, all variables were assessed at a program level rather than an individual horse level. Factors such as temperament, previous experience, workload, and health status are unique to individual horses and may influence a horse’s longevity within a program and reason for retirement. Thus, assessing and tracking such factors at an individual horse level rather than a program level could lead to more meaningful results. Variables reported in this study such as workload and selection procedures may not be good predictors of horse longevity and reason for retirement. Future studies should consider a wider array of variables at an individual horse level.

The authors would suggest changes to the survey instrument before further use. Changing the question on reasons for retiring horses from a ranking question to a percentage response would enhance the data collected and make it easier to analyze data and compare it to other responses collected in the survey. The responses provided to respondents should be defined or respondents should be prompted to define or describe the terms. Additionally, the survey should be updated to reflect current recommendations for optimal terminology in the United States [1]. Providing definitions for the terminology, especially given the recent changes, is recommended. Use of consistent terminology in describing programs, horses, and issues in the industry is crucial to the success of future research.

## 5. Conclusions

Despite the low response rate, this study provides information on the current state of horse selection, retirement, and longevity in EAS helping us understand and characterize this sector of the United States horse industry. Behavior, soundness, and health emerged as important factors in selecting and retiring horses. Future research and education efforts should, therefore, focus on these key areas. A lack of standardization in protocols, practices, and terminology is apparent in the reported data highlighting the need for increased creation and implementation of best practices in addition to those currently recommended by professional organizations. Standardization of terminology and agreement on definitions is needed for future research to be productive. There is also a need for research at an individual horse level, rather than at a program level.

## Figures and Tables

**Table 1:** Number of years member centers and premier accredited centers in Florida have been members of PATH Intl. (formerly NARHA). Data represent number of respondents.

	Member Center	Premier Accredited Center
1–5 years	6	0
6–10 years	2	2
11–15 years	2	0
16–20 years	0	0
21–25 years	3	0
26–30 years	0	1
31–35 years	0	2
36–40 years	0	1

**Table 2:** Training techniques and corresponding descriptions respondents were asked to use in describing their horse training programs. Terms and descriptions are adapted from [14].

Training Technique	Description
Negative Reinforcement	Removal of an aversive stimulus in response to a desired behavior
Positive Reinforcement	Addition of a rewarding stimulus in response to a desired behavior
Negative Punishment	Removal of a rewarding stimulus in response to an undesired behavior
Positive Punishment	Addition of an aversive stimulus in response to an undesired behavior
Systematic Desensitization	Exposure to increasing levels of an arousing stimulus until habituation or a decrease in responsiveness to the stimulus occurs

**Table 3:** Number of responses and response rate by organization.

	Number of Members Invited to Participate	Number of Complete Responses	Response Rate (%)	Number of Incomplete Responses
American Hippotherapy Association (AHA)	2500	70	2.8	75
Professional Association of Therapeutic Horsemanship, International (PATH Intl.)	9000	41	0.5	52
eagala	2000	11	0.6	24
Certified Horsemanship Association (CHA)	12,500	54	0.4	38
Total	26,000	176	0.7	189

**Table 4:** Procedures used in selecting horses and ponies.

	Initial Screening	Pre-Purchase or Donation Exam	Acclimation Period	Trial Period	Other
Frequency [n (%)]	168 (96%)	105 (60%)	147 (84%)	159 (90%)	20 (11%)

**Table 5:** Types of programming offered by AHA, PATH Intl., eagala, and CHA centers.

	AHA	PATH Intl.	eagala	CHA	Total
Adaptive or therapeutic riding [count (%)]	50 (71%)	40 (98%)	5 (46%)	44 (82%)	139 (79%)
Equine assisted physical therapy/hippotherapy [count (%)]	45 (64%)	11 (27%)	3 (27%)	11 (20%)	70 (40%)
Equine assisted occupational therapy/hippotherapy [count (%)]	48 (69%)	10 (24%)	3 (27%)	8 (15%)	69 (39%)
Equine assisted speech therapy/hippotherapy [count (%)]	26 (37%)	4 (10%)	3 (27%)	4 (7%)	37 (21%)
Equine assisted psychotherapy (EAP) or equine facilitated psychotherapy (EFP) [count (%)]	22 (31%)	12 (29%)	8 (73%)	17 (32%)	59 (34%)
Equine assisted learning (EAL) [count (%)]	27 (39%)	21 (51%)	8 (73%)	35 (65%)	91 (52%)
Adaptive driving [count (%)]	8 (11%)	8 (20%)	2 (18%)	7 (13%)	25 (14%)
Interactive vaulting [count (%)]	1 (1%)	2 (5%)	0 (0%)	6 (11%)	9 (5%)
Traditional riding lessons [count (%)]	3 (4%)	1 (2%)	0 (0%)	1 (2%)	5 (3%)
Adaptive unmounted activities [count (%)]	0 (0%)	0 (0%)	1 (9%)	4 (7%)	5 (3%)
Other [count (%)]	4 (6%)	1 (2%)	0 (0%)	2 (4%)	7 (4%)

## Data Availability

The data are not available due to the stipulations provided in the informed consent wherein participants were informed the data would not be made publicly available.

## Appendix A

The survey questions used in Parts I and II of the study are presented in the following tables.

**Table A1:** Survey questions used in part I of the study.

Question	Response Format
Demographics	
What is your role at the center (please select all that apply)? Executive Director Barn or Equine Manager/Equine Specialist Volunteer Coordinator Instructor Volunteer Other (please specify)	Multiple Selection; Open-ended (Other)
2 I am a (please select all that apply) PATH Intl. Registered Level Instructor PATH Intl. Advanced Level Instructor PATH Intl. Master Level Instructor PATH Intl. Interactive Vaulting Instructor PATH Intl. Therapeutic Driving Instructor PATH Intl. Equine Specialist in Mental Health and Learning Speech Therapist Occupational Therapist Physical Therapist Psychotherapist eagala Certified eagala Advanced Certified CHA Certified (please specify certifications held) Other (please specify)	Multiple Selection; Open-ended (CHA Certified & Other)
3 How long has your center been a member of PATH Intl. (previously NARHA)? 1–5 years 6–10 years 11–15 years 16–20 years 21–25 years 26–30 years 31–35 years 36–40 years 40+ years	Single Selection
4 My center is a… PATH Intl. Member Center PATH Intl. Premier Accredited center	Single Selection
5 How many equine related occurrences (human injuries due to equines, for further information refer to standard A24 of the Standards for Certification and Accreditation Manual) are reported each year?	Fill-in-the-blank
6 How many participants does your center serve on a yearly basis?	Fill-in-the-blank
7 Do participants pay for the services offered? Yes No	Single Selection
8 Please list the number of animals that are active in your center’s program. Horses Donkeys Mules Other (please specify)	Fill-in-the-blank; Open-ended (Other)
9 Please list the number of horses that are active in your program and fall within the indicated age range. 1–5 years 6–10 years 11–15 years 16–20 years 21–25 years 26–30 years 30+ years	Fill-in-the-blank
10 On average, how long do horses remain active in your program? 1–5 years 6–10 years 11–15 years 16–20 years 21–25 years 25+ years	Single Selection
11 How many acres of land does your center have access to? 1–5 acres 6–10 acres 11–15 acres 16–20 acres 21–25 acres 25+ acres	Single Selection
Horse Selection and Management	
12 How does your center acquire horses (please select all that apply)? Donation Purchase from trainers and breeders Purchase from private owners Adoption from rescues Free lease Lease Other (please specify)	Multiple Selection; Open-ended (Other)
13 How prepared is your center to… Select and evaluate prospective horses Determine when a horse is ready to retire	Likert-type scale (1 = very prepared, 5 = very unprepared)
14 Does your center have a horse selection protocol? Yes No (skip to question 20)	Single Selection
15 Are you responsible for the horse selection process? Yes No (skip to question 20)	Single Selection
16 Our horse selection protocol includes (please select all that apply): Initial screening Pre-purchase or pre-donation exam by a veterinarian Acclimatization period upon arrival at the center (display question 18) Trial period (display question 19)	Multiple Selection
17 Please list the characteristics you consider when selecting a horse.	Open-ended
18 How long is your center’s acclimatization period? 1–3 days 4–6 days 7–9 days 10–12 days 13–15 days Other (please specify)	Single Selection; Open-ended (Other)
19 How long is your center’s trial period? 1–2 weeks 3–4 weeks 5–6 weeks 7–8 weeks Other (please specify)	Single Selection; Open-ended (Other)
20 Does your center have a set of criteria that are used to determine when horses should be retired? Yes No (skip to question 27)	Single Selection
21 Are you responsible for determining when a horse should be retired? Yes No (skip to question 27)	Single Selection
22 Our retirement protocol takes into account (please select all that apply): Horse behavior during lessons Horse’s interactions with volunteers Horse’s interactions with participants Soundness Overall health Other (please specify)	Multiple Selection; Open-ended (Other)
23 What is the primary reason horses are retired? Unsoundness Behavior (display question 24) Age Other (please specify) (if burnout or burn out, display question 25)	Single Selection; Open-ended (Other)
24 Please describe or list the behaviors that are the reason for retiring horses.	Open-ended
25 Please briefly describe how you know a horse is experiencing burnout.	Open-ended
26 Where do most horses go after they are retired? Retirement Facility Private Owner Previous Owner Stay at Center Other (please specify)	Single Selection; Open-ended (Other)
27 Is your center responsible for the daily care and management of horses? Yes No (skip to question 34)	Single Selection
28 Are you responsible for the daily care and management of horses? Yes No (skip to question 34)	Single Selection
29 In general, what type of turnout is provided to the horses most often? Pasture Small grass lot or paddock Dry lot Indoor arena	Single Selection
30 How many hours per day are horses turned out? Spring; Summer; Fall; Winter Options for each item: 1–4 5–8 9–12 13–16 17–20 20–24	Single Selection
31 Which of the following statements best describes the horses’ social contact with other horses? Horses are turned out with another horse or group of horses Horses have visual and tactile contact with other horses (e.g., horse can touch over a fence line or stall) Horses have visual contact with other horses (e.g., over stall wall, through stall windows) Horses do not have contact with other horses (e.g., housed separately, solid stable walls)	Single Selection
32 On average, our center’s horses have a body condition score of: 1 2 3 4 5 6 7 8 9 I do not know what our center’s horses Body Condition Scores are. I do not know what a Body Condition Score is.	Single Selection
33 Our center has horses that display the following stereotypic behaviors (select all that apply): Cribbing Weaving Stall Walking Stall Kicking Pawing Headshaking Self-mutilation Wood-chewing Other (please specify) Our center does not have horses that display stereotypic behaviors	Multiple Selection; Open-ended (Other)
34 Horses in our program participate in (select all that apply): Therapeutic Riding Hippotherapy Vaulting Driving Equine Assisted Learning Equine Assisted Psychotherapy Riding lessons for riders without disabilities Summer Camps Other (please specify)	Multiple Selection; Open-ended (Other)
35 On average, a horse participates in… EAAT activities ⚬ Hours per day ⚬ Days per week Other Program Activities ⚬ Hours per day ⚬ Days per week	Fill-in-the-blank
36 In addition to program activities, are horses exercised on a regular basis? Yes No (skip to question 40)	Single Selection
37 On average, how many hours of exercise, in addition to program activities, a week do horses receive?	Fill-in-the-blank
38 Who is responsible for exercising horses outside of program activities (please select all that apply)? Instructors Barn Manager Executive Director Volunteer Coordinator Volunteers Other (please specify)	Multiple Selection; Open-ended (Other)
39 How are horses exercised outside of program activities (please select all that apply)? Riding Lunging Hand-walking Ground driving Other (please specify)	Multiple Selection; Open-ended (Other)
40 In addition to program activities and exercise, what activities do your horses participate in on a regular basis (please select all that apply)? Trail rides Free lunging Other (please specify) Our horses only participate in program activities and exercise	Multiple Selection; Open-ended (Other)
41 What type of human-horse interactions, outside of program activities and training, do you conduct with your horses on a regular basis? Grooming outside of preparation for program activities Spending time in the pasture or stall observing or petting the horse Other (please specify) Our horses do not have any human-horse interactions outside of normal program activities and training	Multiple Selection; Open-ended (Other)
Horse and Personnel Training	
42 How prepared is your center to… Train new horses Train horses already in the program Train volunteers in horse handling techniques	Likert-type scale (1 = very prepared, 5 = very unprepared)
43 Does your center have a horse training program that is agreed upon and used by staff? Yes, all staff are required to use and track progress of horses on a computer or using written records Yes, staff sometimes track progress of horses on a computer or written records Yes, staff keep mental notes and report progress verbally to the leadership No, our center does not have a horse training program (skip to question 49) Other (please explain)	Single Selection; Open-ended (Other)
44 Are your responsible for overseeing the horse training program? Yes No (skip to question 49)	Single Selection
45 Please select the choices that best describe your training program. Training utilizing negative reinforcement–removal of something aversive such as pressure to reward a desired response (e.g., applying pressure via a halter and releasing when the horse steps forward); Training utilizing positive reinforcement–addition of something pleasant such as food to reward a desired response (e.g., asking a horse to move forward and scratching their withers when they respond); Training utilizing negative punishment–removal of something pleasant such as food to punish an undesired response; Training utilizing positive punishment–addition of something unpleasant such as a sharp tug on the halter and lead to punish unwanted behavior; Other training method or technique (please specify) Options for each item: Weekly training under saddle Weekly training on the ground Training under saddle when horses display unwanted behaviors Training on the ground when horses display unwanted behaviors	Multiple Selection; Open-ended (Other)
46 Does your center’s horse training program have any challenges? Yes No (skip to question 49)	Single Selection
47 Please briefly describe the challenges and issues associated your center’s horse training program.	Open-ended
48 If provided, would your center utilize instruction regarding horse training techniques and programs? Yes, our center would utilize instruction regarding horse training techniques and programs Our center might utilize instruction regarding horse training techniques and programs No, our center would not utilize instruction regarding horse training techniques and programs No, our center does not need to utilize instruction regarding horse training techniques and programs	Single Selection
49 Does your center utilize the help or services of outside trainers for horse training? We do not use outside trainers (skip to question 50) We occasionally use outside trainers We regularly use outside trainers	Single Selection
50 The help or services of outside trainers are used to (please select all that apply)… Correct unwanted behaviors for horses already in the program Train all new horses entering the program Other (please specify)	Multiple Selection; Open-ended (Other)
51 Do you feel that the equine assisted activities and therapies industry needs better trained horses? Yes, the industry needs better trained horses Yes, some centers need better trained horses No, the industry does not need better trained horses	Single Selection
52 Do you feel that the equine assisted activities and therapies industry needs better horse training programs? Yes, the industry needs better horse training programs Yes, some centers need better horse training programs No, the industry does not need better horse training programs	Single Selection
53 Does your center have a program for training volunteers to be effective horse handlers? Yes No (skip to question 55)	Single Selection
54 Are you responsible for volunteer training? Yes No	Single Selection
55 Does your center offer continuing education to your volunteers? Yes No	Single Selection
56 Does your center offer continuing education for your staff? Yes No	Single Selection
57 Would your center use outside resources (presenters or curriculum) in continuing education for volunteers or staff? Yes, our center would use outside resources in continuing education for staff. (display question 58) Yes, our center would use outside resources in continuing education for volunteers. (display question 58) Yes, our center would use outside resources in continuing education for staff and volunteers. (display question 58) Our center might use outside resources in continuing education for volunteers or staff. (display question 58) No, our center would not use outside resources in continuing education for volunteers or staff.	Single Selection
58 What type of educational resources would you be most likely to utilize (please select all that apply)? Workshop or clinic at your center Off-site clinics or workshops Online courses Curriculum Other (please specify)	Multiple Selection; Open-ended (Other)

**Table A2:** Survey questions used in part II of the study.

Question	Response Format
Our program or center offers (select all that apply): Adaptive or therapeutic riding Equine assisted physical therapy/hippotherapy Equine assisted occupational therapy/hippotherapy Equine assisted speech therapy/hippotherapy Equine assisted psychotherapy (EAP) or equine facilitated psychotherapy (EFP) Equine assisted learning (EAL) Adaptive driving Interactive vaulting Other (please specify):	Multiple Selection; Open-ended (Other)
2 Please list the number of equine animals currently active in your program. Horses, Ponies	Fill-in-the-blank
3 Please indicate the number of horses/ponies in each of the following age ranges. <5 yrs. of age 6–10 yrs. of age 11–15 yrs. of age 16–20 yrs. of age 21–25 yrs. of age 26–30 yrs. of age 31–35 yrs. of age 36–40 yrs. of age 41–45 yrs. of age >45 yrs. of age	Fill-in-the-blank
4 Please indicate the percentage of horses/ponies your program obtains from the sources listed. Donation Adoption from rescue Lease Free Lease Purchase (private owner) Purchase (breeder or trainer) Other (please specify)	Sliding Scale (0–100%); Open-ended (Other)
5 When evaluating horses/ponies, our program utilizes (select all that apply): Initial screening/assessment Pre-purchase/pre-donation exam by a veterinarian Acclimatization period upon arrival at the center Trial period Other (please specify)	Multiple Selection; Open-ended (Other)
6 Please indicate how long horses/ponies remain active in your program. <1 yr. 1–5 yrs. 6–10 yrs. 11–15 yrs. 16–20 yrs. >20 yrs.	Sliding Scale (0–100%)
7 On average, how many days per week do horses/ponies participate in program activities? 1 day/week 2 days/week 3 days/week 4 days/week 5 days/week 6 days/week 7 days/week	Single Selection
8 On average, how many hours per day do horses/ponies participate in program activities? <1 h/day 1–2 h/day 3–4 h/day 5–6 h/day 7–8 h/day 9–10 h/day 11–12 h/day >12 h/day	Single Selection
9 Please rank the reasons for retiring or removing horses/ponies from your program. (1 = most frequent, 7 = least frequent). If a reason is no applicable to your program, please select not applicable. If you have reasons other than those listed, enter them in the “other” text boxes and rank them. If no other reasons are applicable, leave the box empty and select not applicable. Behavior Unsoundness Other health issues Death Other (please specify) Other (please specify) Other (please specify)	Ranking; Open-ended (Other)
